# Review of the health-promoting effects of exercise and the involvement of myokines

**DOI:** 10.20407/fmj.2022-020

**Published:** 2022-12-27

**Authors:** Kazuhiro Nishii, Naoki Aizu, Kouji Yamada

**Affiliations:** 1 Major in Health Sciences, Graduate School of Health Sciences, Fujita Health University, Toyoake, Aichi, Japan; 2 Faculty of Rehabilitation, School of Health Sciences, Fujita Health University, Toyoake, Aichi, Japan

**Keywords:** Myokines, Exercise, Metabolism, Skeletal muscle, Health

## Abstract

Exercise reduces the risk of obesity-based, lifestyle-related diseases, such as metabolic abnormalities and cardiovascular diseases. The present review covers the health-promoting effects of exercise from the point of view of the physiologically active factor myokine, which is secreted by skeletal muscle, and focuses on the skeletal muscle as a new endocrine organ. Myokines have various effects, such as preventing metabolic syndrome by breaking down fat, preventing diabetes by improving glucose metabolism, and preventing progression of arteriosclerosis, dementia, and osteoporosis by enhancing bone metabolism. These substances also stabilize blood pressure, prevent cancer, increase immunity against infections, and prevent the development of age-associated diseases. Myokines are secreted by skeletal muscle into blood vessels, allowing them to exert systemic endocrine effects in organs throughout the body. Myokines are involved in bodily homeostasis and adaptation to the environment, and function by a mechanism similar to that of the skeletal muscle mass regulatory mechanism. Determining the relationships between multiple organs and their biological significance is important for exercise and health research. Progress in this field is expected to result in the identification of pathological mechanisms of action, development of new drugs, evaluation of the effectiveness of biomarkers over a wide range, and future improvement in healthcare.

## Introduction

Exercise is considered to promote good health. However, in concrete terms, it is important to understand how exercise promotes good health. In the current social environment, in which overeating and sedentary lifestyles are common, many people are at risk of developing metabolic syndrome. When more nutrients are not consumed than are metabolized, they accumulate in the body as visceral fat. This fat accumulation contributes to the progression and exacerbation of lifestyle-related diseases, such as arteriosclerosis, which can lead to life-threatening conditions (e.g., myocardial and cerebral infarction). Exercise reduces the accumulation of excess body fat by increasing energy expenditure, and thus alleviates chronic inflammation caused by body fat. However, these positive effects can often be attributed to a reduction in body fat rather than the exercise itself. Indeed, an appropriate body mass can be maintained without exercise, solely by dietary restriction to avoid excessive nutrient intake. In a large-scale epidemiological study, exercise was shown to have various systemic effects, including indirect effects due to fat reduction, and also direct effects, such as maintenance of skeletal muscle mass due to muscular contraction.^1^

## Skeletal muscle as an endocrine organ with secretion of myokines

In recent years, attention has been paid to skeletal muscle as an endocrine organ that secretes various substances. Physiologically active substances secreted by skeletal muscle are collectively termed myokines. The term myokine was coined in 2003 by Pedersen et al., and combines “myo,” meaning “muscle,” and “kine,” meaning “action.”^2^ Myokines are secreted upon skeletal muscle contraction, are transported in the blood throughout the body as autocrine, paracrine, or endocrine substances, and exert effects in distant target organs. Blood concentrations of the myokine interleukin-6 (IL-6) increase during and after exercise. Over 300 myokines have been identified, and new myokines continue to be discovered, but many have unclear or unknown functions.^3^ Those secreted at high concentrations are IL-6, IL-8, C-X-C motif chemokine ligand 1 (CXCL1), leukemia inhibitory factor, colony-stimulating factor 3, IL-1β, and tumor necrosis factor α (TNF-α).^4^ Myokines have various functions and have been studied in various fields, and the mechanism for regulating their secretion is currently being determined (Table 1).

## Activation of myokines and their effects in various organs and tissues

### Suppression of metabolic syndrome: fat breakdown in adipose tissue

The compound β-aminoisobutyric acid (BAIBA) is formed by thymine catabolism. Peroxisome proliferator-activated receptor γ coactivator-1α (PGC-1α) forms complexes with intranuclear receptors and transcription factors, and systematically suppresses expression of genes associated with mitochondrial biosynthesis. In addition, sustained exercise promotes energy metabolism, which leads to an increase in the number of skeletal muscle mitochondria, conversion of muscle fibers to the red muscle type, and capillary angiogenesis. Exercise causes an increase in PGC-1α concentrations, which acts as a trigger for BAIBA secretion into the blood (2–3 μmol/L in humans) from the exercised skeletal muscles. There are two types of adipocytes. White adipocytes accumulate neutral lipids and brown adipocytes use fats as a source of thermal energy. When BAIBA reaches white adipose tissue, it activates expression of thermogenesis via the peroxisome proliferator-activated receptor-α. The process of white fat cells storing fat and brown fat cells burning fat causes white adipocytes to express a gene specific to brown adipocytes and turns white adipose tissue brown.^5^ In addition, BAIBA increases β-oxidation by hepatocytes, induces human multipotent stem cells to develop a phenotype similar to that of brown adipose tissue, and improves glucose homeostasis. In humans, plasma BAIBA concentrations increase with exercise, and high plasma BAIBA concentrations are regarded as a sign of metabolic health. BAIBA may contribute to the protective effects of exercise against metabolic diseases. One result of BAIBA activity is an increase in the basal metabolism of BAIBA-labeled cells. BAIBA has also been suggested to play important roles in cellular metabolism, such as lipid metabolism and regulating insulin, triglyceride, and total cholesterol concentrations.^6^

The myokine irisin is produced when the extracellular domain of the membrane protein fibronectin type III domain-containing protein 5 is severed. Exercise stimulates the secretion of irisin into the blood, where it controls the energy metabolism of adipose tissue.^7^ Increased PGC-1α expression by skeletal muscle due to exercise increases the expression of the thermogenic uncoupling protein 1 (UCP1), thus transforming white adipocytes to brown adipocytes. Additional factors also contribute to the browning of adipocytes. Fibroblast growth factor 21 is secreted by the liver in the fasting state and induces the browning of adipocytes, which is mediated by increased PGC-1α expression in white adipose tissue. In addition, irisin and fibroblast growth factor 21 affect thermogenesis in human brown adipose tissue.^8^ Therefore, myokine-mediated browning of white adipocytes, which facilitates fat metabolism as a thermal energy source, has been clearly shown.

### Prevention of diabetes: improvement of glucose metabolism in the liver and muscles

The secretion of the inflammatory cytokine IL-6 was previously thought to be stimulated by skeletal muscle injury due to excessive contraction. However, *in vitro* studies have shown IL-6 secretion into the blood upon muscle contraction even without associated skeletal muscle injury.^9^ Lipopolysaccharide stimulation of macrophages creates an inflammatory response by increasing TNF-α expression and IL-6 secretion, but IL-6 expression does not increase as a result of exercise.^10^ Increased oxidative stress due to exercise activates nuclear factor κB, which mediates the secretion of IL-6 by myocytes. In addition, when muscular contraction occurs, the release of calcium ions by the muscular endoplasmic reticulum reduces calcium concentrations inside myocytes. Furthermore, this process promotes IL-6 secretion via calcineurin-mediated activation of the nuclear factors of activated T-cells. A decrease in glycogen in skeletal muscle is another factor that stimulates IL-6 secretion.^11^ Exercise and IL-6 secretion are closely linked. In healthy people, IL-6 concentrations increase 100-fold or more after exercise, and are correlated with the duration of exercise.^12^ As a myokine, IL-6 activation of AMP-activated protein kinase is mediated by glycoprotein 130 (CD130), and by increasing insulin signal transmission by skeletal muscle, promotes transfer of glucose transporter 4 to the cell membrane. Additionally, IL-6 activates enzymes associated with β-oxidation of fatty acids, thus increasing fat metabolism in skeletal muscle, and promotes gluconeogenesis in the liver during exercise.^13,14^ Furthermore, as a myokine, IL-6 promotes the secretion of glucagon-like peptide-1 upon exercise. Incretins, which are intestinally secreted insulinotropic compounds, include the gastrointestinal hormone glucagon-like peptide-1 and the glucose-dependent insulinotropic polypeptide. When carbohydrates and fats pass through the intestines, incretins are secreted into the blood by the intestines. Incretins promote insulin secretion and glucagon suppression, thus regulating blood glucose concentrations upon nutrient consumption. Incretins also promote insulin secretion by pancreatic β-cells. Myokines are not only thought to increase insulin sensitivity, but also to have effects on the regulation of insulin secretion by pancreatic β-cells.

Macrophage migration inhibitory factor is involved in glucose metabolism as a myokine. This factor is secreted by skeletal muscles, but only a small proportion enters the blood, where it functions in autocrine fashion. Serine threonine kinase 2 (Akt) is a molecule that is crucial for glucose uptake downstream of the intracellular signal-transmitting insulin receptor. Macrophage migration inhibitory factor has effects on skeletal muscles themselves, suppressing Akt phosphorylation and promoting c-Jun phosphorylation. Macrophage migration inhibitory factor alone does not appear to have effects on glucose uptake, but suppresses insulin-stimulated glucose uptake by skeletal muscles.^15^

### Prevention of arteriosclerosis: suppression of arteriosclerotic progression in vascular walls

Sustained exercise training suppresses the progression of arteriosclerosis. This type of exercise is also associated with the suppression of inflammation and improvement in the blood profiles of lipoproteins, such as low-density lipoprotein and high-density lipoprotein cholesterol. Sustained exercise training increases the expression of PGC-1α, a transcriptional cofactor, in skeletal muscle. Irisin and BAIBA, which are PGC-1α-dependent myokines, have effects such as the browning of white adipose tissue. A study using an arteriosclerosis-prone murine model showed that, although no change was found in the blood lipoprotein profile, which is a risk factor for arteriosclerosis, the area of arteriosclerotic plaques significantly decreased upon sustained exercise training.^16^ In addition, a clear decrease in expression levels of vascular cell adhesion molecule 1 and monocyte chemoattractant protein 1, which induce arteriosclerosis, was observed. The myokines irisin and BAIBA mediated these effects by inhibiting vascular cell adhesion molecule 1 expression in vascular endothelial cells, which suppressed the progression of arteriosclerosis.^16^

Exercise also increases systemic concentrations of numerous cytokines with anti-inflammatory properties.^17^ Several of these cytokines have been identified as humoral factors that are produced in and released by skeletal muscle as myokines.^17–19^ IL-6 has been characterized as a factor that increases the breakdown and oxidation of fat.^20–22^ Acute exercise causes an increase in IL-6 concentrations in the blood, and as they continue to increase, an increase in IL-1Ra and IL-10 concentrations occurs.^23^ Even moderate exercise has major effects on skeletal muscle secretion of IL-6.^24,25^ Furthermore, IL-6 has inhibitory effects on TNF-α and IL-1 production. Taking into consideration that the process of atheromatous arteriosclerosis is a characteristic of vascular inflammation, regular exercise and the consequent increases in concentrations of IL-6 and other myokines may provide indirect protection against diseases associated with mild or chronic systemic inflammation.

### Reducing the risk of dementia onset: effects of myokines on the brain to maintain and improve memory function

In a murine study, cathepsin B knockout mice showed reduced spatial memory. The following study investigated the status of neurogenesis in the hippocampal dentate gyrus while mice were running. In the subventricular zone where there are numerous neural stem cells, doublecortin (a new neuronal marker) only showed a significant increase in type-D cells classified as tanycytes in exercise-burdened, wild-type mice, and neurogenesis was increased in these mice.^26^ Cathepsin B can cross the blood–brain barrier. When mature neural precursor cells were treated with cathepsin B, mRNA and protein expression of neurogenesis-associated doublecortin and brain-derived neurotrophic factor (BDNF) increased. In addition, mature BDNF protein concentrations, mRNA expression of tissue plasminogen activators (t-PA or PLAT), and p11 (a calcium-bound protein) concentrations have been shown to increase in the hippocampus as a result of exercise.^27^ Cathepsin B, which is mediated by p11, activates neurogenesis in the hippocampal dentate gyrus, contributing to the maintenance and improvement of memory function.^26^

In 5XFAD (B6SJL) mice, which model Alzheimer’s disease, amyloid β starts to accumulate in the brain approximately 6 weeks after birth, and memory impairment is present at approximately 16 weeks of age. Hindleg atrophy was induced in some mice by fitting them with a plaster cast for 2 weeks at the age of 12 weeks (i.e., before the usual onset of memory impairment). These mice developed memory impairment at a younger age than 5XFAD mice that never wore a cast. The atrophied muscles of these mice showed elevated hemopexin levels, which were also increased in the blood and hippocampus. Therefore, effects of hemopexin on these mice may have occurred after it reached the brain via the bloodstream. Hemopexin is a protein that binds heme. Hemopexin is released into the blood and then circulates around the entire body in the bloodstream. When hemopexin was administered continuously for 2 weeks directly into the ventricles of mice with Alzheimer’s disease that were 6 to 7 weeks old, and therefore were too young to develop dementia, memory impairment developed.^28^ Lipocalin 2, which is a factor associated with neuroinflammation in the murine brain, also increased. Therefore, the onset of memory impairment occurred earlier in mice with skeletal muscle atrophy, and this was due to the effects of hemopexin on the brain secreted by atrophied muscles.

Decreased muscle strength has also recently been shown to increase the risks of cognitive impairment and depression. A group of human subjects with low muscle strength had a higher annual risk of developing mild cognitive impairment than a group with high muscle strength.^29^ Exercise improves the cerebral circulation, increases neuronal activity, and improves brain performance. In the long term, insufficient exercise reduces serotonin secretion, generally leading to excessive stress. Insufficient exercise also leads to a decrease in sleep quality and delayed recovery from fatigue. Therefore, the maintenance of skeletal muscle not only maintains exercise performance and protects internal organs, but is also associated with the maintenance of cerebral and cardiac health.

### Prevention of a decrease in bone mineral density: promotion of bone formation involving muscle mass control and an increase in bone metabolism

There are direct interactions between skeletal muscle and bone. An exercise-related load on bone maintains and increases bone mass. Therefore, a quantitative and qualitative decrease in skeletal muscle associated with sarcopenia may also lead to a decrease in bone mass. There are several myokines that affect bone mass.^30^ Myostatin, also known as growth differentiation factor 8, is a representative myokine that belongs to the transforming growth factor β (TGFβ) superfamily and negatively controls skeletal muscle mass. TGFβ expression is suppressed by exercise. Sarcopenia involves promotion of that negative effect, with suppression of osteoblast proliferation and promotion of osteoclast differentiation.^31^ Follistatin binds with myostatin, preventing it from binding to receptors, and thus positively controls bone mass.^32^ In addition, IL-6 increases the expression of receptor activator of nuclear factor κB ligand and matrix metalloproteinase, thus increasing bone resorption.^33^ Similarly, IL-7, which is an inflammatory cytokine, increases the expression of receptor activator of nuclear factor κB ligand.^34^ Bone morphogenetic protein 7, which is a member of the TGFβ superfamily, promotes cartilage and bone formation, and is also involved in muscular hypertrophy.^35^

Insulin-like growth factor-1 (IGF-1) has various functions, such as increasing glucose uptake mediated by a signal transmission molecule in a manner similar to the mechanism of action of insulin, promoting the synthesis of and suppressing the breakdown of glycogen, and increasing the uptake of free fatty acids. In addition, by promoting protein synthesis and cell proliferation, IGF-1 promotes an increase in muscle mass.^36^ IGF-1 is present in systemically circulating and localized forms. The localized form of IGF-1 occurs as the three isoforms of IGF-1Ea, b, and c. Among these, IGF-1Ec, also termed “mechano growth factor,” is secreted as a result of skeletal muscular contraction, contributes to the maintenance of and an increase in muscle mass, and works with other isoforms to positively control bone mass.^37^ In a study using an ovariectomized murine model, we found that bone remodeling in the femur and lumbar vertebrae was activated as a result of stimulation by exercise. Furthermore, exercise has been shown to increase skeletal muscle IL-6, IL-10, and IL-15 expression, and to suppress TNF-α expression.^38–41^

### Blood pressure stability: direct or indirect blood pressure regulation

Apelin is a physiologically active peptide that is secreted by myocytes and adipocytes, and promotes glucose energy metabolism^42^ and insulin sensitivity.^43^ Apelin is an endogenous ligand of the apelin receptor (APJ), which is an orphan G-protein-coupled receptor.^44,45^ Apelin generates nitric oxide by activating endothelial nitric oxide synthase (eNOS) present in vascular endothelial cells and has hypotensive effects due to vasodilation.^46,47^ The receptors APJ and angiotensin II type-1 receptor bind together to form a heterodimer. These receptors function as an endogenous antagonistic system that suppresses the angiotensin II type 1 receptor hypertensive system, with an endogenous suppressive mechanism of hypertensive activity.^47,48^

Furthermore, apelin is expressed in tissues, such as adipose tissue, the kidneys, the heart, and blood vessels, as well as skeletal muscle.^49,50^ Apelin induces an aortal increase in eNOS phosphorylation activity.^47^ Obesity reduces eNOS phosphorylation activity in the arteries, and the consequent decreased nitric oxide production exacerbates arteriosclerosis.^51,52^ Vasodilation due to increased arterial nitric oxide leads to reduced arteriosclerosis.^52^ Additionally, apelin plays a role in promoting nitric oxide production by binding to APJ in endothelial cells, thus promoting eNOS phosphorylation mediated by AMP-activated protein kinase or by Akt from phosphoinositide 3-kinase.^53–55^

In vascular tissue, APJ is expressed by endothelial cells and smooth myocytes, and endothelial APJ induces vasodilation, which reduces blood pressure.^47^ However, APJ may also have vasoconstrictive activity.^56,57^ With stimulation by apelin, the APJ ligand increases blood pressure, constricts blood vessels, and with simultaneous stimulation of the APJα1A adrenalin-receptor, strongly constricts isolated blood vessels.

### Prevention of cancer: suppression of cancer cell proliferation due to humoral immunity

There have been numerous reports regarding the potential for the suppression of cancer proliferation by exercise. In a study in which mice were injected with cancer cells, cancer lesion growth rates were compared between groups with and without exercise.^58^ Cancer growth was found to be suppressed in the exercise group, and the mechanism involved promoting the mobilization of natural killer cells mediated by epinephrine and IL-6.^59^

Myokines are thought to be responsible for some of the various mechanisms of suppressing cancer growth due to exercise, and are considered to have cancer-growth-suppressing effects involving secreted protein acidic and rich in cysteine (SPARC).^60^ SPARC levels decrease with aging and inactivity.^60^ Although skeletal muscles are present throughout the body, cancer metastasis to the muscles is uncommon, and antioxidants and enzymes present in myocytes might be important for suppressing cancer growth.

In a study using a murine azoxymethan-induced colorectal cancer model with SPARC-knockout, low-intensity treadmill exercise three times each week for 6 weeks markedly suppressed aberrant crypt focus formation in wild-type mice. However, this model did not suppress crypt focus formation in SPARC-knockout mice. Additionally, while the apoptosis-positive cell count was significantly higher in the exercise group than in the resting group in wild-type mice, it was significantly lower in SPARC-knockout mice, with no changes due to exercise. Active caspase 3 and caspase 8, which are apoptosis-related proteins, increased with exercise in wild-type mice, but did not increase in SPARC-knockout mice. In addition, when exogenous SPARC was administered, aberrant crypt focus formation was suppressed in a concentration-dependent manner. As a myokine, SPARC has the potential to contribute to the preventive effects of exercise on colorectal cancer by inducing apoptosis. This contribution may involve apoptosis-promoting effects mediated by caspase 8 and caspase 3 activation.^60^

### Prevention of infection: immune cell activation and an increase in resistance

IL-15 is a 4α helical protein and a T-cell proliferating factor with numerous biological characteristics. IL-15 has approximately 19% sequence homology with IL-2. Large quantities of IL-15 are produced in skeletal muscle and the placenta,^61^ and its expression levels in skeletal muscles are second only to those of IL-6.^62^ IL-15 is also important for the differentiation of natural killer cells.^63^ In addition, CD8^+^CD122^+^ T cells, which express the IL-2-receptor β-chain, proliferate in response to IL-15 stimulation. Therefore, IL-15 is a regulatory factor with important roles in maintaining functional homeostasis. In mice, CD8^+^CD122^+^ T cell counts increase in an age-dependent manner and show high interferon-γ productivity. Therefore, T cells are considered to be involved in bacterial elimination after bacterial infection.

IL-8, also known as CXCL-8, is a CXC chemokine. Chemokines are cytokines for which neutrophils display chemotaxis. IL-8 is a typical inflammatory chemokine that is thought to play an important role during exercise. IL-8 is produced by various cells in the body, but is primarily produced at sites of inflammation and is involved in neutrophil migration. IL-8 concentrations in the blood increase during exercise.^62,64,65^ IL-8 is a myokine, and its production during exercise is also induced by skeletal muscle. Additionally, the release of IL-8 produced in skeletal muscle is considered to result in increased IL-8 blood concentrations.^66^

CXCL1 is produced by fibroblasts, epithelial cells, and monocytes under conditions of tissue damage or infection. CXCL1 is mainly involved in wound healing and the promotion of migration of neutrophils and other cells of leukocyte lineage. CXCL1 is a myokine that shows increased expression after skeletal muscular contraction and exercise, but its levels do not increase owing to exercise-related inflammation.

Decreased blood IL-15 concentrations are associated with the exacerbation of chronic obstructive pulmonary disease.^67^ Chronic obstructive pulmonary disease involves an aging-related decrease in muscle mass, and patients enter a sarcopenic state with decreased grip strength and muscular strength throughout the body, notably in the leg and torso muscles. The development of sarcopenia is considered to lead to decreased IL-5 myokine levels, which are thought to be associated with decreased pulmonary immune function in chronic obstructive pulmonary disease.

## Myokine function

### Downregulation of myokine expression by exercise stimulation

Multifaceted research has shown increased myokine expression due to stimulation by exercise. However, myokines can also show decreased expression due to exercise. A typical myokine that is suppressed by exercise is myostatin, but there have been recent reports of other exercise-suppressed myokines, such as CCL5 and CXCL10. CXCL10 acts as a strong angiogenesis-suppressing factor. Therefore, an exercise-induced decrease in CXCL10 secretion promotes angiogenesis. Vascular function decreases in association with aging and the onset of diabetes, and exercise prevents this decrease in vascular function. An exercise-dependent decrease in CCL5 expression, especially in white muscle, has been observed.^68,69^

### Molecules other than proteins and peptides also act as myokines

There have been reports of molecules other than proteins and peptides acting as myokines. BAIBA, which is a metabolite of the amino acid valine, which is secreted by skeletal muscles, increases brown fat levels and promotes lipid metabolism.^5^ Furthermore, an increase in the blood concentration of kynurenine, which is a tryptophan metabolite, is correlated with depression. Prolonged exercise increases the levels of enzymes that transform kynurenine to kynurenic acid in skeletal muscle, and reduces the secretion of kynurenine by skeletal muscle, which may promote the alleviation of depression.^70^ These findings have led to the view of myokines as including low-molecular-weight compounds in addition to proteins and peptides.

Decorin proteins comprise a family of small leucine-rich proteoglycans that interact with type-1 collagen fibers. These interactions influence the dynamics of collagen fiber formation and the distances between adjacent collagen fibers. The binding of decorin by numerous cell surface receptors results in the stimulation of autophagy and inflammation, and suppressive effects on angiogenesis and tumor formation, which are involved in tumor suppression. In addition, decorin binds with myostatin, which is a factor that suppresses skeletal muscle differentiation and growth, and thus suppresses those activities. Blood decorin concentrations increase with muscular strength training. Studies on decorin-overexpressing mice have shown that muscular differentiation marker levels increase, whereas the expression of ubiquitin ligands, which are associated with muscular atrophy, is suppressed.^71^

### Adipokines are bioactive substances that are similar to myokines and secreted by adipocytes

Adipocytes are not solely for energy storage, and attention has recently been paid to their role in secreting myokine-like physiologically active substances, collectively termed “adipokines.” Adipokines include “bad” adipokines, such as TNF-α, plasminogen activator inhibitor-1, and heparin-binding epidermal growth factor-like growth factor, which promote arteriosclerosis; and “good” adipokines, which have the opposite, protective effect. Surplus energy due to overeating is stored in adipocytes, and as they increase in size, they secrete less adiponectin (health-promoting molecules in adipokines). In particular, in metabolic syndrome, an increase in visceral fat leads to abnormalities in blood glucose concentrations, blood pressure, and lipid metabolism, and adiponectin secretion decreases. In addition, adiponectin increases insulin activity and suppresses the formation of lipid abnormalities and chronic inflammation. Attention has recently been paid to the relationship between dementia and fatty liver. Myonectin is a myokine that is also known as complement C1q tumor necrosis factor-related protein.^15^ Upon entering a starvation state, autophagy starts in the liver. If intracellular recycling of amino acids and other molecules increases to obtain sufficient nutrients, autophagy is suppressed. Myonectin from skeletal muscle decreases in the starvation state and increases under conditions of sufficient nutrition. Studies using cultured cells have shown that myonectin suppresses autophagy in hepatocytes, with mediation by the phosphoinositide 3-kinase/Akt mammalian target of the rapamycin system.^72^

## Discussion

Myokines were initially defined as cytokines that are produced in and secreted by skeletal muscle, and exert physiological effects at other sites in the body.^2^ However, myokines were later found to not all be cytokines, and they include metabolites and low-molecular-weight molecules other than proteins and peptides. Therefore, the term “myokine” has been accepted as a general term for substances that are secreted in connection with skeletal muscle contraction, and that are transported as autocrine or paracrine substances or hormones to distant target organs where they exert endocrine effects. Numerous myokines have been discovered in the course of comprehensive analyses, but the functions of many have not been fully determined. As a result, the term “myokine” is not used as a comprehensive term describing substances secreted due to exercise or muscular contraction, but rather for describing physiologically active factors secreted by skeletal muscle. Muscular hypertrophy and contraction are not required to meet this definition, and some myokines are not proteins.

**Figure 1:**
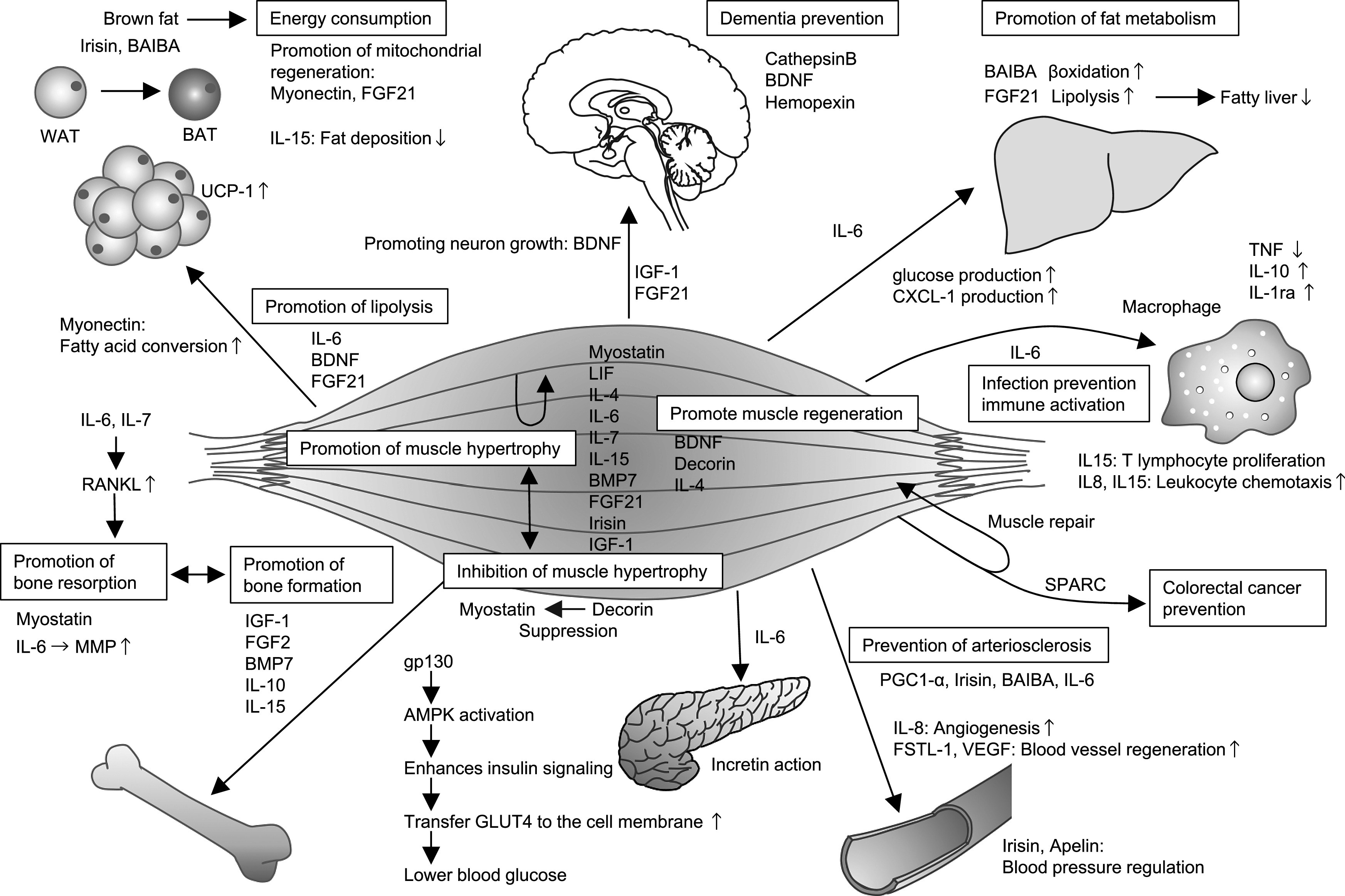
Activities of skeletal muscle as an endocrine organ The activities of myokines in numerous organs and tissues, and the resulting effects are shown. Myokines are secreted by skeletal muscle. Autocrine myokines that promote muscular hypertrophy and regeneration are shown in the center of skeletal muscle. In particular, we referred to Reference, which shows the action of myokine in various fields.^74,75^ FSTL-1: follistatin-like 1; VEGF: vascular endothelial growth factor; UCP-1: uncoupling protein 1; BAT: brown adipose tissue; WAT: white adipose tissue.

Since the concept of myokines was first presented,^2^ the activities of various physiologically active substances secreted by skeletal muscle have been identified. These have been suggested to be involved in bodily homeostasis and adaptation to the environment.^73^ Furthermore, most myokines share a molecular basis with the skeletal mass regulatory mechanism, and analysis of the biological importance of a multiorgan association with skeletal muscle as a single node is currently a field of intense research. To date, the principal roles of myokines include the alleviation of fatty liver and the breakdown of body fat by promoting metabolism, improvement of glucose metabolism, and prevention of diabetes (Figure 1).^74,75^ Additionally, myokines prevent dementia, promote bone formation and prevent a decrease in bone mineral density, prevent arteriosclerosis, stabilize blood pressure, prevent infection by increasing immune strength, prevent age-related diseases, and have anti-inflammatory effects. Hopefully, progress in studies of myokines will result in the determination of the pathological mechanisms of action, development of new drugs and evaluation of biomarker effectiveness over a wide range, and future improvement in healthcare.

## Figures and Tables

**Table1:** Myokine and functions

	function	Myokine
Suppression of metabolic syndrome	Work on adipose tissue to break down fat	BAIBA PGC-1α Irisin
Diabetes prevention	Improves glucose metabolism by acting on the liver and muscles	IL-6 MIF
Prevention of arteriosclerosis	Acts on the walls of blood vessels and suppresses the progression of arteriosclerosis	PGC-1α Irisin BAIBA IL-6
Reduces the risk of developing dementia	Acts on the brain to maintain and improve memory function	CathepsinB BDNF Hemopexin
Prevention of bone density loss	Controls muscle mass, enhances bone metabolism and promotes bone formation	Myostatin Follistatin IL-6 IL-7 IL-10 IL-15 IGF-1
Blood pressure stability	Directly or indirectly regulate blood pressure	Apelin
Cancer prevention	Suppression of cancer cell proliferation by humoral immunity	SPARC IL-6
Infection prevention	Activation of immune cells and improvement of resistance	IL-15 IL-8

## References

[1] Lee IM, Shiroma EJ, Lobelo F, Puska P, Blair SN, Katzmarzyk PT. Effect of physical inactivity on major non-communicable diseases worldwide: An analysis of burden of disease and life expectancy. Lancet 2012; 380: 219–229. 22818936 10.1016/S0140-6736(12)61031-9 PMC3645500

[2] Pedersen BK, Steensberg A, Fischer C, Keller C, Keller P, Plomgaard P, Febbraio M, Saltin B. Searching for the exercise factor: Is IL-6 a candidate? J Muscle Res Cell Motil 2003; 24: 113–119. 14609022 10.1023/a:1026070911202

[3] Hartwig S, Raschke S, Knebel B, et al. Secretome profiling of primary human skeletal myocytes. Biochim Biophys Acta 2014; 1844: 1011–1017. 23994228 10.1016/j.bbapap.2013.08.004

[4] Scheler M, Irmler M, Lehr S, Hartwig S, Staiger H, Al-Hasani H, Beckers J, de Angelis MH, Häring HU, Weigert C. Cytokine response of primary human myotubes in an in vitro exercise model. Am J Physiol Cell Physiol 2013; 305: C877–C886. 23926130 10.1152/ajpcell.00043.2013

[5] Roberts LD, Boström P, O’Sullivan JF, et al. β-Aminoisobutyric acid induces browning of white fat and hepatic β-oxidation and is inversely correlated with cardiometabolic risk factors. Cell Metab 2014; 19: 96–108. 24411942 10.1016/j.cmet.2013.12.003 PMC4017355

[6] Roberts LD, Boström P, O’Sullivan JF, et al. β-Aminoisobutyric acid induces browning of white fat and hepatic β-oxidation and is inversely correlated with cardiometabolic risk factors. Cell Metab 2014; 19: 96–108. 24411942 10.1016/j.cmet.2013.12.003 PMC4017355

[7] Boström P, Wu J, Jedrychowski MP, et al. A PGC1-α-dependent myokine that drives brown-fat-like development of white fat and thermogenesis. Nature 2012; 481: 463–468. 22237023 10.1038/nature10777 PMC3522098

[8] Lee P, Linderman JD, Smith S, Brychta RJ, Wang J, Idelson C, Perron RM, Werner CD, Phan GQ, Kammula US, Kebebew E, Pacak K, Chen KY, Celi FS. Irisin and FGF21 are cold-induced endocrine activators of brown fat function in humans. Cell Metab 2014; 19: 302–309. 24506871 10.1016/j.cmet.2013.12.017 PMC7647184

[9] Steensberg A, van Hall G, Osada T, Sacchetti M, Saltin B, Klarlund Pedersen B. Production of interleukin-6 in contracting human skeletal muscles can account for the exercise-induced increase in plasma interleukin-6. J Physiol 2000; 529 Pt 1: 237–242. 10.1111/j.1469-7793.2000.00237.x PMC2270169 11080265

[10] Ullum H, Haahr PM, Diamant M, Palmø J, Halkjaer-Kristensen J, Pedersen BK. Bicycle exercise enhances plasma IL-6 but does not change IL-1 alpha, IL-1 beta, IL-6, or TNF-alpha pre-mRNA in BMNC. J Appl Physiol (1985) 1994; 77: 93–97. 10.1152/jappl.1994.77.1.93 7961280

[11] Steensberg A, Febbraio MA, Osada T, Schjerling P, van Hall G, Saltin B, Pedersen BK. Interleukin-6 production in contracting human skeletal muscle is influenced by pre-exercise muscle glycogen content. J Physiol 2001; 537: 633–639. 11731593 10.1111/j.1469-7793.2001.00633.x PMC2278951

[12] Fischer CP. Interleukin-6 in acute exercise and training: What is the biological relevance? Exerc Immunol Rev 2006; 12: 6–33. 17201070

[13] Carey AL, Steinberg GR, Macaulay SL, Thomas WG, Holmes AG, Ramm G, Prelovsek O, Hohnen-Behrens C, Watt MJ, James DE, Kemp BE, Pedersen BK, Febbraio MA. Interleukin-6 increases insulin-stimulated glucose disposal in humans and glucose uptake and fatty acid oxidation in vitro via AMP-activated protein kinase. Diabetes 2006; 55: 2688–2697. 17003332 10.2337/db05-1404

[14] Bruce CR, Dyck DJ. Cytokine regulation of skeletal muscle fatty acid metabolism: Effect of interleukin-6 and tumor necrosis factor-alpha. Am J Physiol Endocrinol Metab 2004; 287: E616–E621. 15149950 10.1152/ajpendo.00150.2004

[15] Fujii NL, Manabe Y, Furuichi Y. Macrophage Migration Inhibitory Factor Regulates Glucose Metabolism in Skeletal Muscle. Descente Sports Science 2016; 37: 3–9.

[16] Shimba Y, Togawa H, Senoo N, Ikeda M, Miyoshi N, Morita A, Miura S. Skeletal Muscle-specific PGC-1α Overexpression Suppresses Atherosclerosis in Apolipoprotein E-Knockout Mice. Sci Rep 2019; 9: 4077. 30858489 10.1038/s41598-019-40643-1 PMC6411944

[17] Petersen AM, Pedersen BK. The anti-inflammatory effect of exercise. J Appl Physiol (1985) 2005; 98: 1154–1162. 10.1152/japplphysiol.00164.2004 15772055

[18] Pedersen BK, Febbraio M. Muscle-derived interleukin-6—a possible link between skeletal muscle, adipose tissue, liver, and brain. Brain Behav Immun 2005; 19: 371–376. 15935612 10.1016/j.bbi.2005.04.008

[19] Pedersen BK, Steensberg A, Fischer C, Keller C, Keller P, Plomgaard P, Wolsk-Petersen E, Febbraio M. The metabolic role of IL-6 produced during exercise: Is IL-6 an exercise factor? Proc Nutr Soc 2004; 63: 263–267. 15294041 10.1079/PNS2004338

[20] Lyngsø D, Simonsen L, Bülow J. Interleukin-6 production in human subcutaneous abdominal adipose tissue: The effect of exercise. J Physiol 2002; 543: 373–378. 12181307 10.1113/jphysiol.2002.019380 PMC2290472

[21] Petersen EW, Carey AL, Sacchetti M, Steinberg GR, Macaulay SL, Febbraio MA, Pedersen BK. Acute IL-6 treatment increases fatty acid turnover in elderly humans in vivo and in tissue culture in vitro. Am J Physiol Endocrinol Metab 2005; 288: E155–E162. 15383370 10.1152/ajpendo.00257.2004

[22] Hall G, Steensberg A, Sacchetti M, Fischer C, Keller C, Schjerling P, Hiscock N, Møller K, Saltin B, Febbraio MA, Pedersen BK. Interleukin-6 stimulates lipolysis and fat oxidation in humans. J Clin Endocrinol Metab 2003; 88: 3005–3010. 12843134 10.1210/jc.2002-021687

[23] Steensberg A, Fischer CP, Keller C, Møller K, Pedersen BK. IL-6 enhances plasma IL-1ra, IL-10, and cortisol in humans. Am J Physiol Endocrinol Metab 2003; 285: E433–E437. 12857678 10.1152/ajpendo.00074.2003

[24] Fischer CP, Hiscock NJ, Penkowa M, Basu S, Vessby B, Kallner A, Sjöberg LB, Pedersen BK. Supplementation with vitamins C and E inhibits the release of interleukin-6 from contracting human skeletal muscle. J Physiol 2004; 558: 633–645. 15169848 10.1113/jphysiol.2004.066779 PMC1664960

[25] Pedersen M, Steensberg A, Keller C, Osada T, Zacho M, Saltin B, Febbraio MA, Pedersen BK. Does the aging skeletal muscle maintain its endocrine function? Exerc Immunol Rev 2004; 10: 42–55. 15633585

[26] Moon HY, Becke A, Berron D, Becker B, Sah N, Benoni G, Janke E, Lubejko ST, Greig NH, Mattison JA, Duzel E, van Praag H. Running-Induced Systemic Cathepsin B Secretion Is Associated with Memory Function. Cell Metab 2016; 24: 332–340. 27345423 10.1016/j.cmet.2016.05.025 PMC6029441

[27] Sartori CR, Vieira AS, Ferrari EM, Langone F, Tongiorgi E, Parada CA. The antidepressive effect of the physical exercise correlates with increased levels of mature BDNF, and proBDNF proteolytic cleavage-related genes, p11 and tPA. Neuroscience 2011; 180: 9–18. 21371535 10.1016/j.neuroscience.2011.02.055

[28] Nagase T, Tohda C. Skeletal muscle atrophy-induced hemopexin accelerates onset of cognitive impairment in Alzheimer’s disease. J Cachexia Sarcopenia Muscle 2021; 12: 2199–2210. 34658156 10.1002/jcsm.12830 PMC8718090

[29] Boyle PA, Buchman AS, Wilson RS, Leurgans SE, Bennett DA. Association of muscle strength with the risk of Alzheimer disease and the rate of cognitive decline in community-dwelling older persons. Arch Neurol 2009; 66: 1339–1344. 19901164 10.1001/archneurol.2009.240 PMC2838435

[30] Guo B, Zhang ZK, Liang C, Li J, Liu J, Lu A, Zhang BT, Zhang G. Molecular communication from skeletal muscle to bone: A review for muscle-derived myokines regulating bone metabolism. Calcif Tissue Int 2017; 100: 184–192. 27830278 10.1007/s00223-016-0209-4

[31] Dankbar B, Fennen M, Brunert D, Hayer S, Frank S, Wehmeyer C, Beckmann D, Paruzel P, Bertrand J, Redlich K, Koers-Wunrau C, Stratis A, Korb-Pap A, Pap T. Myostatin is a direct regulator of osteoclast differentiation and its inhibition reduces inflammatory joint destruction in mice. Nat Med 2015; 21: 1085–1090. 26236992 10.1038/nm.3917

[32] Amthor H, Nicholas G, McKinnell I, Kemp CF, Sharma M, Kambadur R, Patel K. Follistatin complexes Myostatin and antagonises Myostatin-mediated inhibition of myogenesis. Dev Biol 2004; 270: 19–30. 15136138 10.1016/j.ydbio.2004.01.046

[33] Benedetti FD, Rucci N, Fattore AD, Peruzzi B, Paro R, Longo M, Vivarelli M, Muratori F, Berni S, Ballanti P, Ferrari S, Teti A. Impaired skeletal development in interleukin-6-transgenic mice: A model for the impact of chronic inflammation on the growing skeletal system. Arthritis Rheum 2006; 54: 3551–3563. 17075861 10.1002/art.22175

[34] Weitzmann MN, Roggia C, Toraldo G, Weitzmann L, Pacifici R. Increased production of IL-7 uncouples bone formation from bone resorption during estrogen deficiency. J Clin Invest 2002; 110: 1643–1650. 12464669 10.1172/JCI15687 PMC151629

[35] Winbanks CE, Chen JL, Qian H, Liu Y, Bernardo BC, Beyer C, Watt KI, Thomson RE, Connor T, Turner BJ, McMullen JR, Larsson L, McGee SL, Harrison CA, Gregorevic P. The bone morphogenetic protein axis is a positive regulator of skeletal muscle mass. J Cell Biol 2013; 203: 345–357. 24145169 10.1083/jcb.201211134 PMC3812980

[36] Zoncu R, Efeyan A, Sabatini DM. mTOR: From growth signal integration to cancer, diabetes and ageing. Nat Rev Mol Cell Biol 2011; 12: 21–35. 21157483 10.1038/nrm3025 PMC3390257

[37] Tanaka K, Matsumoto E, Higashimaki Y, Katagiri T, Sugimoto T, Seino S, Kaji H. Role of osteoglycin in the linkage between muscle and bone. J Biol Chem 2012; 287: 11616–11628. 22351757 10.1074/jbc.M111.292193 PMC3320911

[38] Yao R, Nishii K, Kito T, Teranishi T, Sugiyama T, Sakai K, Matsubara M, Yamada K. A novel device to prevent osteoporosis by promoting bone metabolism using a newly developed double-loading stimulation with vibration and shaking. Okajimas Folia Anat Jpn 2019; 96: 13–21. 31462620 10.2535/ofaj.96.13

[39] Kito T, Nishii K, Yao R, Teranishi T, Sugiyama T, Sakai K, Matsubara M, Yamada K. Effect of shaking and vibration stimulation on lumbar vertebrae in ovariectomized mice. Fujita Med J 2019; 5: 57–62. 35111503 10.20407/fmj.2018-006 PMC8766245

[40] Yamada K, Nishii K, Sakai K, Teranishi T. Stimulus in the form of rotation and shaking of a platform and its effect on the formation of trabecular bone in the lumbar vertebrae of mice. Aging Clin Exp Res 2013; 25: 625–632. 24146364 10.1007/s40520-013-0164-0

[41] Kito T, Teranishi T, Nishii K, Sakai K, Matsubara M, Yamada K. Effectiveness of exercise-induced cytokines in alleviating arthritis symptoms in arthritis model mice. Okajimas Folia Anat Jpn 2016; 93: 81–88. 28216540 10.2535/ofaj.93.81

[42] Dray C, Knauf C, Daviaud D, Waget A, Boucher J, Buléon M, Cani PD, Attané C, Guigné C, Carpéné C, Burcelin R, Castan-Laurell I, Valet P. Apelin stimulates glucose utilization in normal and obese insulin-resistant mice. Cell Metab 2008; 8: 437–445. 19046574 10.1016/j.cmet.2008.10.003

[43] Yue P, Jin H, Aillaud M, Deng AC, Azuma J, Asagami T, Kundu RK, Reaven GM, Quertermous T, Tsao PS. Apelin is necessary for the maintenance of insulin sensitivity. Am J Physiol Endocrinol Metab 2010; 298: E59–E67. 19861585 10.1152/ajpendo.00385.2009 PMC2806109

[44] Tatemoto K, Hosoya M, Habata Y, Fujii R, Kakegawa T, Zou MX, Kawamata Y, Fukusumi S, Hinuma S, Kitada C, Kurokawa T, Onda H, Fujino M. Isolation and characterization of a novel endogenous peptide ligand for the human APJ receptor. Biochem Biophys Res Commun 1998; 251: 471–476. 9792798 10.1006/bbrc.1998.9489

[45] O’Dowd BF, Heiber M, Chan A, Heng HH, Tsui LC, Kennedy JL, Shi X, Petronis A, George SR, Nguyen T. A human gene that shows identity with the gene encoding the angiotensin receptor is located on chromosome 11. Gene 1993; 136: 355–360. 8294032 10.1016/0378-1119(93)90495-o

[46] Tatemoto K, Takayama K, Zou MX, Kumaki I, Zhang W, Kumano K, Fujimiya M. The novel peptide apelin lowers blood pressure via a nitric oxide-dependent mechanism. Regul Pept 2001; 99: 87–92. 11384769 10.1016/s0167-0115(01)00236-1

[47] Ishida J, Hashimoto T, Hashimoto Y, et al. Regulatory roles for APJ, a seven-transmembrane receptor related to angiotensin-type 1 receptor in blood pressure in vivo. J Biol Chem 2004; 279: 26274–26279. 15087458 10.1074/jbc.M404149200

[48] Chun HJ, Ali ZA, Kojima Y, Kundu RK, Sheikh AY, Agrawal R, Zheng L, Leeper NJ, Pearl NE, Patterson AJ, Anderson JP, Tsao PS, Lenardo MJ, Ashley EA, Quertermous T. Apelin signaling antagonizes Ang II effects in mouse models of atherosclerosis. J Clin Invest 2008; 118: 3343–3354. 18769630 10.1172/JCI34871 PMC2525695

[49] Kleinz MJ, Davenport AP. Emerging roles of apelin in biology and medicine. Pharmacol Ther 2005; 107: 198–211. 15907343 10.1016/j.pharmthera.2005.04.001

[50] Kleinz MJ, Skepper JN, Davenport AP. Immunocytochemical localisation of the apelin receptor, APJ, to human cardiomyocytes, vascular smooth muscle and endothelial cells. Regul Pept 2005; 126: 233–240. 15664671 10.1016/j.regpep.2004.10.019

[51] Ma L, Ma S, He H, Yang D, Chen X, Luo Z, Liu D, Zhu Z. Perivascular fat-mediated vascular dysfunction and remodeling through the AMPK/mTOR pathway in high-fat diet-induced obese rats. Hypertens Res 2010; 33: 446–453. 20186150 10.1038/hr.2010.11

[52] Fujie S, Hasegawa N, Kurihara T, Sanada K, Hamaoka T, Iemitsu M. Association between aerobic exercise training effects of serum adropin level, arterial stiffness, and adiposity in obese elderly adults. Appl Physiol Nutr Metab 2017; 42: 8–14. 27897440 10.1139/apnm-2016-0310

[53] Hu H, He L, Li L, Chen L. Apelin/APJ system as a therapeutic target in diabetes and its complications. Mol Genet Metab 2016; 119: 20–27. 27650065 10.1016/j.ymgme.2016.07.012

[54] Busch R, Strohbach A, Pennewitz M, Lorenz F, Bahls M, Busch MC, Felix SB. Regulation of the endothelial apelin/APJ system by hemodynamic fluid flow. Cell Signal 2015; 27: 1286–1296. 25817266 10.1016/j.cellsig.2015.03.011

[55] Yang X, Zhu W, Zhang P, Chen K, Zhao L, Li J, Wei M, Liu M. Apelin-13 stimulates angiogenesis by promoting cross‑talk between AMP-activated protein kinase and Akt signaling in myocardial microvascular endothelial cells. Mol Med Rep 2014; 9: 1590–1596. 24573187 10.3892/mmr.2014.1984

[56] Hashimoto T, Kihara M, Ishida J, Imai N, Yoshida S, Toya Y, Fukamizu A, Kitamura H, Umemura S. Apelin stimulates myosin light chain phosphorylation in vascular smooth myocytes. Arterioscler Thromb Vasc Biol 2006; 26: 1267–1272. 16556853 10.1161/01.ATV.0000218841.39828.91

[57] Nagano K, Ishida J, Unno M, Matsukura T, Fukamizu A. Apelin elevates blood pressure in ICR mice with L‑NAME‑induced endothelial dysfunction. Mol Med Rep 2013; 7: 1371–1375. 23525196 10.3892/mmr.2013.1378 PMC3658861

[58] Pedersen L, Idorn M, Olofsson GH, Lauenborg B, Nookaew I, Hansen RH, Johannesen HH, Becker JC, Pedersen KS, Dethlefsen C, Nielsen J, Gehl J, Pedersen BK, Thor Straten P, Hojman P. Voluntary Running Suppresses Tumor Growth through Epinephrine- and IL-6-Dependent NK Cell Mobilization and Redistribution. Cell Metab 2016; 23: 554–562. 26895752 10.1016/j.cmet.2016.01.011

[59] Idorn M, Hojman P. Exercise-dependent regulation of NK cells in cancer protection. Trends Mol Med 2016; 22: 565–577. 27262760 10.1016/j.molmed.2016.05.007

[60] Aoi W, Naito Y, Takagi T, et al. A novel myokine, secreted protein acidic and rich in cysteine (SPARC), suppresses colon tumorigenesis via regular exercise. Gut 2013; 62: 882–889. 22851666 10.1136/gutjnl-2011-300776

[61] Grabstein KH, Eisenman J, Shanebeck K, Rauch C, Srinivasan S, Fung V, Beers C, Richardson J, Schoenborn MA, Ahdieh M, Johnson L, Alderson MR, Watson JD, Anderson DM, Giri JG. Cloning of a T cell growth factor that interacts with the beta chain of the interleukin-2 receptor. Science 1994; 264: 965–968. 8178155 10.1126/science.8178155

[62] Nieman DC, Davis JM, Henson DA, Walberg-Rankin J, Shute M, Dumke CL, Utter AC, Vinci DM, Carson JA, Brown A, Lee WJ, McAnulty SR, McAnulty LS. Carbohydrate ingestion influences skeletal muscle cytokine mRNA and plasma cytokine levels after a 3-h run. J Appl Physiol (1985) 2003; 94: 1917–1925. 10.1152/japplphysiol.01130.2002 12533503

[63] Kennedy MK, Glaccum M, Brown N, et al. Reversible defects in natural killer and memory CD8 T cell lineages in interleukin 15-deficient mice. J Exp Med 2000; 191: 771–780. 10704459 10.1084/jem.191.5.771 PMC2195858

[64] Nieman DC, Henson DA, Smith LL, Utter AC, Vinci DM, Davis JM, Kaminsky DE, Shute M. Cytokine changes after a marathon race. J Appl Physiol (1985) 2001; 91: 109–114. 10.1152/jappl.2001.91.1.109 11408420

[65] Suzuki K, Nakaji S, Yamada M, Liu Q, Kurakake S, Okamura N, Kumae T, Umeda T, Sugawara K. Impact of a competitive marathon race on systemic cytokine and neutrophil responses. Med Sci Sports Exerc 2003; 35: 348–355. 12569227 10.1249/01.MSS.0000048861.57899.04

[66] Pedersen BK, Akerström TC, Nielsen AR, Fischer CP. Role of myokines in exercise and metabolism. J Appl Physiol (1985) 2007; 103: 1093–1098. 10.1152/japplphysiol.00080.2007 17347387

[67] Han MK, Quibrera PM, Carretta EE, et al. Frequency of exacerbations in patients with chronic obstructive pulmonary disease: An analysis of the SPIROMICS cohort. Lancet Respir Med 2017; 5: 619–626. 28668356 10.1016/S2213-2600(17)30207-2 PMC5558856

[68] Ishiuchi Y, Sato H, Tsujimura K, Kawaguchi H, Matsuwaki T, Yamanouchi K, Nishihara M, Nedachi T. Skeletal muscle cell contraction reduces a novel myokine, chemokine (C-X-C motif) ligand 10 (CXCL10): Potential roles in exercise-regulated angiogenesis. Biosci Biotechnol Biochem 2018; 82: 97–105. 29235416 10.1080/09168451.2017.1411778

[69] Ishiuchi Y, Sato H, Komatsu N, Kawaguchi H, Matsuwaki T, Yamanouchi K, Nishihara M, Nedachi T. Identification of CCL5/RANTES as a novel contraction-reducible myokine in mouse skeletal muscle. Cytokine 2018; 108: 17–23. 29558694 10.1016/j.cyto.2018.03.012

[70] Arem H, Moore SC, Patel A, Hartge P, Berrington de Gonzalez A, Visvanathan K, Campbell PT, Freedman M, Weiderpass E, Adami HO, Linet MS, Lee IM, Matthews CE. Leisure time physical activity and mortality: A detailed pooled analysis of the dose-response relationship. JAMA Intern Med 2015; 175: 959–967. 25844730 10.1001/jamainternmed.2015.0533 PMC4451435

[71] Kanzleiter T, Rath M, Görgens SW, Jensen J, Tangen DS, Kolnes AJ, Kolnes KJ, Lee S, Eckel J, Schürmann A, Eckardt K. The myokine decorin is regulated by contraction and involved in muscle hypertrophy. Biochem Biophys Res Commun 2014; 450: 1089–1094. 24996176 10.1016/j.bbrc.2014.06.123

[72] Seldin MM, Lei X, Tan SY, Stanson KP, Wei Z, Wong GW. Skeletal muscle-derived myonectin activates the mammalian target of rapamycin (mTOR) pathway to suppress autophagy in liver. J Biol Chem 2013; 288: 36073–36082. 24187137 10.1074/jbc.M113.500736 PMC3861655

[73] Whitham M, Febbraio MA. The ever-expanding myokinome: Discovery challenges and therapeutic implications. Nat Rev Drug Discov 2016; 15: 719–729. 27616294 10.1038/nrd.2016.153

[74] Pedersen BK, Febbraio MA. Muscles, exercise and obesity: Skeletal muscle as a secretory organ. Nat Rev Endocrinol 2012; 8: 457–465. 22473333 10.1038/nrendo.2012.49

[75] Severinsen MCK, Pedersen BK. Muscle-Organ Crosstalk: The Emerging Roles of Myokines. Endocr Rev 2020; 41: 594–609. 32393961 10.1210/endrev/bnaa016 PMC7288608

